# Case Report: Two Patients with Partial DiGeorge Syndrome Presenting with Attention Disorder and Learning Difficulties

**DOI:** 10.4274/jcrpe.v3i2.19

**Published:** 2011-06-08

**Authors:** Bülent Hacıhamdioğlu, Merih Berberoğlu, Zeynep Şıklar, Figen Doğu, Pelin Bilir, Şenay Savaş Erdeve, Aydan İkincioğulları, Gönül Öçal

**Affiliations:** 1 Ankara University School of Medicine, Department of Pediatric Endocrinology, Cebeci, Ankara, Turkey; 2 Ankara University School of Medicine, Department of Pediatric Immunology, Cebeci, Ankara, Turkey; +90 312 595 67 91+90 312 319 14 40hacihamdi@mynet.comAnkara University School of Medicine, Department of Pediatric Endocrinology, Cebeci, Ankara, Turkey

**Keywords:** DiGeorge syndrome, psychiatric problems, adolescent

## Abstract

DiGeorge syndrome (DGS) has classically been characterized by the triad of clinical features including congenital cardiac defects, immune deficiencies secondary to aplasia or hypoplasia of the thymus, and hypocalcaemia due to small or absent parathyroid glands. The phenotypic features of these patients are much more variable and extensive than previously ecognized. The acknowledgement of similarities and phenotypic overlap of DGS with other disorders associated with genetic defects in 22q11 has led to an expanded description of the phenotypic features of DGS including palatal/speech abnormalities, as well as cognitive, neurological and psychiatric disorders. Here, we report the cases of two DGS patients with dysmorphic facial features who were initially admitted to the Psychiatry Department for attention disorder and learning difficulties.

**Conflict of interest:**None declared.

## INTRODUCTION

Introduction

DiGeorge syndrome (DGS) is one of the most common chromosomal disorders, with an estimated prevalence of 1 in 4 000-6 000 live births (1,2). It is actually a well-defined primary immunodeficiency disorder classically associated with abnormal facial appearance, congenital heart defects, hypoparathyroidism with hypocalcaemia, as well as cognitive, behavioral and psychiatric problems. Pathological hallmarks include conotruncal abnormalities and absence or hypoplasia of the thymus and parathyroid glands (3,4). Depending on whether thymic hypoplasia or aplasia is present, DGS can be classified as partial or complete. The term complete DGS is used to describe patients with DGS who are athymic and have no circulating T cells; this group constitutes less than 1% of all DGS cases. Patients with partial DGS have thymic hypoplasia that is evidenced by the presence of circulating T cells (5).

The facial appearance of patients with DGS is characterized by hypertelorism, micrognathia, short philtrum with fish-mouth appearance, antimongoloid slant, and telecanthus with short palpebral fissures (3,4). However, not all patients with DGS show these typical dysmorphic findings and the diagnosis can be delayed for many years (6). It has been reported that children and adults with DGS have high rates of behavioral, psychiatric and communication disorders (7).

Children and adolescents with subtle dysmorphic findings can be admitted to a psychiatric department initially before being diagnosed with DGS. Here, we report the cases of two children who demonstrated similar features of partial DGS and were initially admitted to a psychiatric department prior to the exact diagnosis.

## PATIENT 1

**Figure 1:**
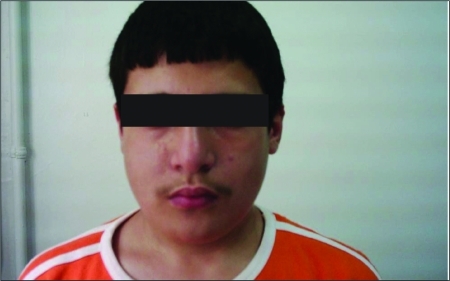
Prominent nose with a bulbous tip, small mouth and eyes, ocular hypertelorism, and long face

Patient 1 was a 13.6-year-old boy born to unrelated healthy parents. Initially, he was admitted to the Psychiatry Department with attention deficit and socialization problems. Following psychiatric assessment, the patient was referred to our pediatric endocrinology department for evaluation of possible hypothyroidism. The patient’s mother reported that he was inattentive and asocial. The boy had medical history of mild recurrent numbness in his hands in the past 2 years. At physical examination, mild facial dysmorphism was noted - prominent nose with a bulbous tip, small mouth and eyes, ocular hypertelorism, and long face (Figure 1). The laboratory investigations revealed a serum calcium level of 6.6 mg/dL, phosphorus 8 mg/dL, intact parathormone level of 24.9 pg/mL (normal: 10-65), 25-hydroxyvitamin D level of 25 μg/L, and urine calcium/creatinine ratio of 0.01. His thyroid hormone levels were within the reference range. Despite the normal parathormone levels, the presence of hypocalcemia and hyperphosphatemia suggested a possible diagnosis of hypoparathyroidism. The thymus was not visualized at scintigraphic evaluation. Immunologic studies revealed normal levels (%) and absolute numbers (per mm3) of T cells/T-cell subsets, B cells and NK cells, except for a slightly decreased CD3+ (CD16+56+)-cell level (48-58%; normal range for children: 58-82%). The lymphoproliferative response to both phytohaemagglutinin (PHA) and anti-CD3 monoclonal antibodies was also found to be normal. There was no clinically overt immunodeficiency. The patient had no cardiac defects. Ultrasonography of the left kidney demonstrated a cortical cyst (Table 1). Fluorescence in situ hybridization (FISH) analysis, using the Tuple probe specific to the DGS region, showed the chromosome 22q11.2 deletion.

**Figure 2:**
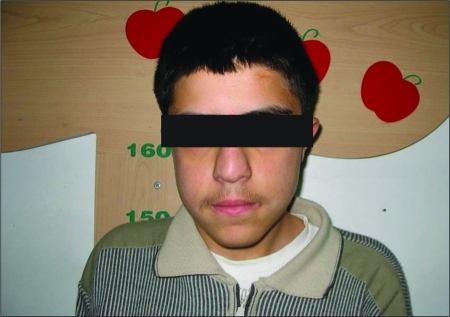
Similar phenotype - prominent nose with a bulbous tip, small mouth and eyes, ocular hypertelorism, and long face

Patient 2 was a 14.2-year-old boy born to unrelated parents. He presented to the Psychiatry Department with learning difficulties and numbness in his hands before being admitted to our pediatric endocrinology department. He was referred to our clinic for evaluation of a possible diagnosis of hypothyroidism and hypocalcaemia. The patient’s medical history revealed impaired school performance and mild recurrent numbness in his hands. He had an atrial septal defect (ASD) repaired when he was 6 years old. Physical examination showed mild facial dysmorphism with prominent nose and bulbous tip, small mouth and eyes, ocular hypertelorism, and long face (Figure 2). Chvostek’s and Trousseau’s signs were positive. The laboratory investigations revealed hypoparathyroidism. Serum calcium level was 4.6 mg/dL, phosphorus 8.4 mg/dL, parathormone level was normal (32.9 pg/mL, reference range: 10-65 pg/mL), 25-hydroxyvitamin D level was 31 μg/L, and urine calcium/creatinine ratio was 0.02. The patient was euthyroid. The thymus was not visualized by thoracic computed tomography. Immunologic studies showed low levels of CD3+ and CD8+ T lymphocytes. There was no clinically overt immunodeficiency (Table 1). Peripheral blood CD3, CD4I, CD8, CD19 and CD16+56+ cell counts in absolute numbers, as well as the lymphoproliferative response to PHA and anti-CD3 monoclonal antibodies,  were found to be normal. FISH analysis revealed chromosome 22q11.2 deletion.

**Table 1:**
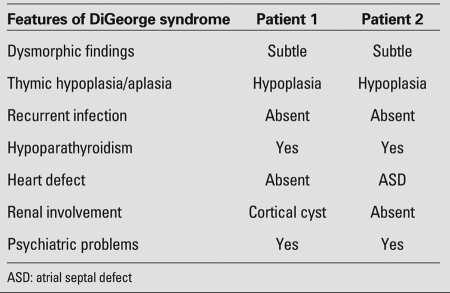
Findings related to DiGeorge syndrome in the two patients

## DISCUSSION

The specific FISH test for chromosome 22q11 deletion is the standard method for diagnosis of DGS. The wide availability of commercial FISH probes has enhanced the clinicians’ ability to diagnose and treat the affected children rapidly (8). 22q11 deletion has been demonstrated by FISH method in both of our patients. Patients with chromosome 22q11.2 deletion do not always show all components of DGS. Hypoparathyroidism can be the only abnormality and may exist with no accompanying cardiac or immunologic defects (9). Also, in children and adolescents, hypoparathyroidism due to DGS may not always show typical hypocalcemic symptoms. Neither of our patients exhibited any symptoms of hypoparathyroidism, except numbness. 

DGS patients do not always have the typical dysmorphic features and may not be diagnosed until adulthood. The characteristic facial and otolaryngic findings  of this syndrome are hypertelorism, micrognathia, a short philtrum with a fish-mouth appearance, small hooded eyes, antimongoloid slant, telecanthus with short palpebral fissures, low-set ears often with defective pinnae, and submucous cleft palate (3,4). Both our patients had a prominent nose with a bulbous tip, small mouth and eyes, ocular hypertelorism, and long face. Patients with clinical features associated with DGS, such as symptoms of hypoparathyroidism, immune deficiency or cardiac defects, should be evaluated for silent dysmorphologic features, especially those affecting the morphology of the nose, mouth, and eyes. Furthermore, children and adolescents with DGS frequently have affective, anxiety, attention deficit and behavioral disorders. DGS can be associated with severe psychopathology in adults, including bipolar disorder and schizophrenia. Cognitive disabilities are seen in 40 to 46% of individuals with 22q11.2 deletion and the majority of them are mild to moderate (10). For this reason, it is possible for patients with undiagnosed DGS to first be admitted to a psychiatry department.  Both of our patients had psychiatric symptoms and initially presented to the Psychiatry Department. DGS should be considered in patients who have dysmorphic face and history suggesting DGS (recurrent infections, numbness and cardiopathy).

The severity of immunodeficiency varies among DGS patients. Most have a mild form characterized by a small, histologically normal thymus, low absolute T-cell numbers and normal or near-normal T-cell functions. No clinical and laboratory evidence of significant immunodeficiency was found in our patients and they were diagnosed with partial DGS. Cardiac defects are well-recognized abnormalities of the 22q11.2 deletion syndrome, reported in 75% of patients (4). Our first patient had no cardiac abnormality, whereas the second one had an ASD.

Facial dysmorphic findings associated with DGS can also be overlooked if the clinician is not well acquainted with these features. DGS is relatively common and this diagnosis should be considered in patients who have mental, behavioral, or attention disorder accompanied by silent dysmorphic features.
